# Diagnosis of Chronic Pulmonary Aspergillosis: Which Is the Best Investigation?

**DOI:** 10.4269/ajtmh.23-0053a

**Published:** 2023-04-17

**Authors:** Inderpaul Singh Sehgal, Ritesh Agarwal

**Affiliations:** Department of Pulmonary Medicine Postgraduate Institute of Medical Education and Research (PGIMER) Chandigarh, India E-mail: inderpgi@outlook.com; Department of Pulmonary Medicine Postgraduate Institute of Medical Education and Research (PGIMER) Chandigarh, India E-mail: agarwal.ritesh@outlook.in

Dear Editor,

We read the interesting article by de Oliveira et al. on the performance of various investigations for diagnosing chronic pulmonary aspergillosis (CPA).^1^ The authors conclude that the diagnostic performance of the currently available tests is poor. Unfortunately, the authors have not measured serum *Aspergillus fumigatus*-specific IgG in their cohort by other more sensitive methods. Previous studies have demonstrated that detection of IgG antibodies by immunoassays is more sensitive than by immunoprecipitation methods (double immunodiffusion [DID] method or counterimmunoelectrophoresis [CIE] method).^2^^–^^4^ We found that for every six tests done with the DID method, one additional diagnosis of allergic bronchopulmonary aspergillosis (ABPA) was secured when *A.fumigatus*-IgG was detected using the immunoassay. Notably, it is not clear against which reference standard the authors compared the performance of the index test (DID, CIE, serum or bronchoalveolar lavage fluid galactomannan index [GMI], or histopathology). For example, if DID or CIE was used to diagnose CPA, how could the performance of DID or CIE be assessed?^5^ The authors found histology to have a sensitivity of 78%. To our understanding, histology is the current reference standard and should be able to diagnose CPA in all cases. Finally, a receiver operating curve analysis was not performed to ascertain the diagnostic performance of the tests evaluated in the current study. The cut-off values of serum and BALF GMI used by the authors may not have been correct. In a recent prospective study, we found that serum and BALF GMI had the best sensitivities at cut-offs of 0.6 and 1.4, respectively, for diagnosing CPA.^6^ Importantly, we diagnosed CPA using a composite of clinical, radiological, and microbiological (culture positivity for aspergillus in respiratory secretions or *A.fumigatus*-specific IgG [fluorescent enzyme immunoassay method, FEIA]) assessments.^6^ Future studies should use either point-of-care tests or automated methods to detect *A.fumigatus*-specific IgG and should also develop cut-off values specific to their population.^7^
